# Efficacy of Sorafenib Monotherapy versus Sorafenib-Based Loco-Regional Treatments in Advanced Hepatocellular Carcinoma

**DOI:** 10.1371/journal.pone.0077240

**Published:** 2013-10-14

**Authors:** Sangheun Lee, Beom Kyung Kim, Seung Up Kim, Yehyun Park, Sooyun Chang, Jun Yong Park, Do Young Kim, Sang Hoon Ahn, Chae Yoon Chon, Kwang-Hyub Han

**Affiliations:** 1 Department of Internal Medicine, Yonsei University College of Medicine, Seoul, Republic of Korea; 2 Institute of Gastroenterology, Yonsei University College of Medicine, Seoul, Republic of Korea; 3 Liver Cancer Special Clinic, Yonsei University College of Medicine, Seoul, Republic of Korea; 4 Liver Cirrhosis Clinical Research Center, Seoul, Republic of Korea; 5 Brain Korea 21 Project for Medical Science, Seoul, Republic of Korea; Davidoff Center, Israel

## Abstract

**Background:**

Although sorafenib is accepted as the standard of care in advanced hepatocellular carcinoma (HCC), its therapeutic benefit is marginal. Here, we aimed to compare the efficacy and safety of sorafenib monotherapy (S-M) and sorafenib-based loco-regional treatments (S-LRTs) in advanced HCC.

**Methods:**

From 2007 to 2012, 290 patients with advanced HCC (Barcelona Clinic Liver Cancer stage C) with S-M (n = 226) or S-LRTs (n = 64) were reviewed retrospectively. Survival outcomes and treatment-related toxicities between two groups were analyzed.

**Results:**

Variables related to tumor burden and liver function were similar between the groups (all *P* > 0.05). Within the entire population, the S-LRTs group had both longer median overall survival (OS) (8.5 vs 5.5 months, *P* = 0.001) and progression-free survival (PFS) (5.3 vs 3.0 months, *P* = 0.002) than the S-M group. Furthermore, the S-LRTs group had longer Os than the S-M group in a subgroup with neither extrahepatic spread (EHS) nor regional nodal involvement (RNI) (18.0 vs 7.8 months, *P* = 0.019) and in a subgroup with EHS and/or RNI (8.3 vs 4.8 months, *P* = 0.028). In addition, the S-LRTs group had longer PFS than the S-M group in the subgroup with neither EHS nor RNI (9.6 vs 3.2 months, *P* = 0.027).

**Treatment:**

Related toxicity was similar between two groups.

**Conclusion:**

Combined use of sorafenib and LRTs may provide better treatment outcomes without significantly increasing treatment-related toxicities, even in patients with EHS and/or RNI. Therefore, addition of active LRTs might be considered, if feasible.

## Introduction

Hepatocellular carcinoma (HCC) is currently ranked as the ﬁfth most common cancer and the third leading cause of cancer death worldwide [1]. Surgical resection or local ablative treatments such as radiofrequency ablation and percutaneous ethanol injection achieve the best outcomes, with a 5-year survival rate of 60–70% in patients treated during early stages. Unfortunately, this is not feasible for the majority of patients with HCC, because they present with advanced disease, consisting of extensive tumor burden with portal vein thrombosis, intra/extra tumor spread, or poor liver function [2,3].

 To date, several palliative therapeutic options have been used to treat advanced HCC. These include trans-arterial chemoembolization (TACE), hepatic artery infusional chemotherapy (HAIC), external/internal irradiation, and molecular targeted agents [4-6]. Of these, sorafenib, a small-molecule multi-targeted tyrosine kinase inhibitor, is the ﬁrst targeted agent approved for the systemic treatment of advanced HCC. Sorafenib inhibits vascular endothelial growth factor receptor, platelet-derived growth factor receptor, B-Raf, Fms-related tyrosine kinase, and c-kit at nanomolar concentrations [7,8]. Further, sorafenib demonstrates survival benefits when compared to the best supportive care [5,6] and has thus become the principal therapy for patients with advanced HCC or Barcelona Clinic Liver Cancer (BCLC) stage C. It is also currently being used in the control arm of ongoing clinical trials for new targeted agents [9-12]. Although sorafenib is the standard treatment for advanced HCC, most patients treated with sorafenib achieve only stable disease as the best radiologic response, with a median time to progression of from 2.2 to, at most, 5.5 months. More importantly, its therapeutic benefit might be substantially attenuated for patients with portal vein invasion and/or extrahepatic spread. Thus, these patients will have a much poorer overall survival compared to those without these two factors [13,14].

 Therefore, additional treatment strategies need to be identified and optimized to improve the therapeutic response. Furthermore, given that more than two-thirds of patients with advanced HCC die of liver failure due to intrahepatic tumor progression rather than from progression of extrahepatic metastatic disease, loco-regional treatments (LRTs) targeting the primary tumor should be reappraised from the standpoint of therapeutic benefit for patients with advanced HCC. Indeed, before sorafenib treatment was widely accepted for advanced HCC, several researchers had investigated this issue and reported some benefits in patients with major vascular invasion or extrahepatic metastasis where LRTs were used to delay intra-hepatic tumor progression instead of the best supportive care [9,10,15,16]. More recently, several studies with small population have suggested that LRTs when combined with sorafenib may lead to better clinical outcomes than the traditional sorafenib-monotherapy (S-M) [17-20]. 

 Here, we aimed to assess the efficacy and safety of the combined use of sorafenib and LRTs in advanced HCC and compare these outcomes to those achieved with S-M.

## Materials and Methods

### Patient Eligibility

Between December 2007 and February 2012 a total of 318 patients with unresectable HCC who were eligible for sorafenib treatment with or without LRTs were reviewed retrospectively. Among patients who underwent S-LRT or SM, 28 patients were excluded according to the following exclusion criteria; short duration of sorafenib administration (<2 week, n = 21), BCLC stage A/B (n = 5), Child-Pugh class C (n = 2), and Eastern Cooperative Oncology Group (ECOG) performance status ≥3 (n = 0). Finally, 290 patients with BCLC C and Child-Pugh class A or B were analyzed. 

 This study was performed in accordance with the ethical guidelines of the 1975 Declaration of Helsinki. Written informed consent was obtained from each participant or responsible family member after possible complications of the diagnostic procedures and anti-cancer treatments had been fully explained. This study was approved by the independent institutional review board of Severance Hospital.

### Diagnosis of HCC

A diagnosis of HCC was based on histological examination or clinico-radiologic criteria according to guidelines proposed by The Korea Liver Cancer Study Group [21] as follows; a patient is considered positive for HCC if they have one or more risk factors (hepatitis B or C virus infection, cirrhosis) and one of the following: 1) serum α-fetoprotein (AFP) ≥ 400 ng/mL and a positive finding on at least one of three typical imaging studies [dynamic computed tomography (CT), dynamic magnetic resonance imaging (MRI), or hepatic angiography], or 2) serum AFP <400 ng/mL and positive findings on at least two of the three imaging studies. A positive finding for typical HCC on dynamic CT or MRI was defined as increased arterial enhancement followed by decreased enhancement compared with the liver (washout) in the portal or equilibrium phase.

### Study Design

Subjects were divided into two groups; 1) patients who were treated with sorafenib monotherapy (referred to as “S-M group”), and 2) patients who were treated by other LRTs in addition to sorafenib (referred to as “S-LRTs group”). Patients in the S-M group received 400 mg oral sorafenib (Nexavar Bayer Health Care AG, Leverkusen, Germany) twice daily on a continuous dosing schedule. In addition to the twice daily 400 mg oral sorafenib, patients in the S-LRTs group also received LRTs that included intra-arterial chemotherapy (either TACE or HAIC), external beam radiotherapy, or both to delay progression of intra-hepatic tumors. The detailed protocols of LRTs performed at our institution, such as TACE using doxorubicin, lipiodol, and gelatin sponge particles, HAIC via implantable port system using 5-fluorouracil and cisplatin, and three-dimensional conformal external beam radiotherapy were described previously [22-25]. S-LRTs were performed in advanced HCC with the tolerable liver function for anti-tumor therapy in Severance. However, S-M has been applied for patients who couldn’t afford S-LRTs because national insurance system didn’t guarantee only one method among chemotherapy and LRTs.

 The time interval between LRTs and initiation of sorafenib is less than 14 days (median 4 days, range 0–14 days). In both groups, dose reduction and drug interruption were allowed in the case of significant drug-related adverse effects or poor general condition. Sorafenib was also administered until disease progression, the development of intolerable toxicities, or patient refusal. However, for patients in the S-LRTs group, when intrahepatic tumor progression was observed, an alternative LRTs was allowed to delay intrahepatic disease progression, according to physician discretion and patient eligibility.

### Evaluation of Treatment Response and Safety Profile

Clinical examination and laboratory assessments were performed upon the initiation of sorafenib treatment. The response evaluation was carried out with a dynamic CT scan or MRI, if appropriate, every 8 weeks. We adopted the modified Response Evaluation Criteria in Solid Tumors as follows: complete response (CR), partial response (PR), stable disease (SD), and progressive disease (PD). Objective response was defined as CR or PR, and disease control as CR, PR, or SD. Response was analyzed by intention-to-treat analysis. Toxicity grade was assessed before each treatment cycle using the National Cancer Institute Common Toxicity Criteria version 3.0

### Statistical Analyses

Student’s *t* test or Mann-Whitney *U* tests, if appropriate, were used to compare continuous variables, and Chi-square or Fisher’s exact tests were used for categorical variables. The primary endpoint was overall survival (OS), whereas the secondary endpoints were progression-free survival (PFS) and treatment-related toxicity. OS was calculated as the time interval from the initiation date of either sorafenib or LRTs (provided that LRTs preceded the administration of sorafenib in the S-LRTs group) until the date of death or final follow-up. Similarly, PFS was calculated as the time interval from the initiation date of sorafenib or LRTs until the date of first progression or death. Survival time was estimated by the Kaplan-Meier method, and the survival difference between groups was assessed by the log-rank test. The Cox proportional hazards model was used for a multivariate analysis of survival. All variables found significant in the univariate analysis were included in the multivariate model. Statistical analyses were performed using SAS version 9.1.3 (SAS, Cary, NC). A two-sided *P* value < 0.05 was considered statistically significant.

## Results

### Baseline Characteristics

Table 1 shows the baseline characteristics of the entire population. The median age was 56.7 years and 243 (83.8%) patients were male. Most patients (n = 208, 71.7 %) showed preserved liver function of Child-Pugh class A. The median tumor size was 4.0 cm. Extrahepatic spreading (EHS) was identified in 182 (62.8%) patients, whereas regional lymph nodal involvement (RNI) was noted in 89 (36.0%) patients. The most common site for EHS was lung (n = 132, 45.5%). The median AFP and protein induced by vitamin K absence or antagonist (PIVKA) levels were 110 ng/mL and 112 AU/L, respectively. In S-LRTs group, sorafenib was combined with LRTs as follows; TACE (n = 24), HAIC (n = 5), HAIC with radiotherapy (n = 13), and radiotherapy alone (n = 22). The proportion of patients with a previous history of treatment for HCC and non-viral etiology was higher in the S-M group than in the S-LRTs group (79.6% vs. 67.2%, *P* = 0.037 and 15.9% vs. 6.2%, *P* = 0.047, respectively). There was no statistically significant difference in clinical variables between the two groups (Table 1).

**Table 1:** Baseline characters of patients in entire cohort.

**Variables**		**Entire population (n = 290)**	**S-M group (n= 226, 77.9%)**	**S-LRTs group (n = 64, 22.1%)**	***P***
Age (years)		56.7 (27.0–85.0)	57.0 (27.0–85.0)	56.0 (28.0–79.0)	NS
Male gender		243 (83.8)	188 (83.2)	55 (85.9)	NS
Etiology, viral/ non-viral		250 (86.2)/40 (13.8)	190 (84.1)/36 (15.9)	60 (93.8)/4 (6.2)	0.047
ECOG, 0/1~2		84 (29.0)/206 (71.0)	63 (27.9)/163 (72.1)	21 (32.8)/43 (67.2)	NS
Child-Pugh class, A/B		208(71.7)/82 (28.3)	161 (71.2)/65 (28.8)	47 (73.4)/17 (26.6)	NS
Tumor size (cm)		4.0 (0.7–20.0)	4.0 (1.0–20.0)	4.7 (0.7–16.0)	NS
Presence of EHS		182 (62.8)	147 (65.0)	35 (54.7)	NS
Presence of lung metastasis		132 (45.5)	109 (48.2)	23 (35.9)	0.081
Presence of bone metastasis		44 (15.2)	39 (17.3)	5 (7.8)	0.063
Presence of distant LN metastasis		26 (9.0)	17 (7.5)	9 (14.1)	NS
Presence of RNI		89 (30.7)	66 (29.2)	23 (35.9)	NS
Prior history of HCC treatment^¶^		223 (76.9)	180 (79.6)	43 (67.2)	0.037
	Surgery	17	13	4	
	RFA	2	2	0	
	PEIT	0	0	0	
	TACE	97	71	26	
	TACE with RFA	4	1	3	
	TACE with radiotherapy	9	9	0	
	HAIC with radiotherapy	86	78	8	
	HAIC	5	4	1	
	Radiotherapy	3	2	1	
AFP, ng/mL		110 (0.9–120,000.0)	231.3 (1.1–120,000.0)	160.5 (0.9–83,000.0)	NS
PIVKA, AU/L		112 (6.2–75,000.0)	733.0 (6.2–75,000.0)	329.5 (10.0–2,000.0)	0.098
Ln total dosage (mg		10.7 (5.9–13.4)	10.7 (5.9–13.4)	10.9 (8.5–12.8)	NS

Abbreviations: S-M, sorafenib monotherapy; S-LRTs, sorafenib combined with loco-regional treatments; ECOG, Eastern Cooperative Oncology Group; HCC, hepatocellular carcinoma; EHS, extrahepatic spread; LN, lymph node; RNI, regional nodal involvement; RFA, radiofrequency ablation; PEIT, percutaneous ethanol injection therapy; TACE, trans-arterial chemoembolization; HAIC, hepatic arterial infusional chemotherapy; AFP, α-fetoprotein; PIVKA, protein induced by vitamin K absence; Ln, natural logarithm; NS, not significant.

Values are expressed as median (range) or no. (%).

^¶^ If different anti-cancer treatment modalities were performed repeatedly before enrollment, the latest treatment modality was recorded as the “prior history of HCC treatment”.

### Treatment Outcomes and Variables Affecting OS

**Figure 1:**
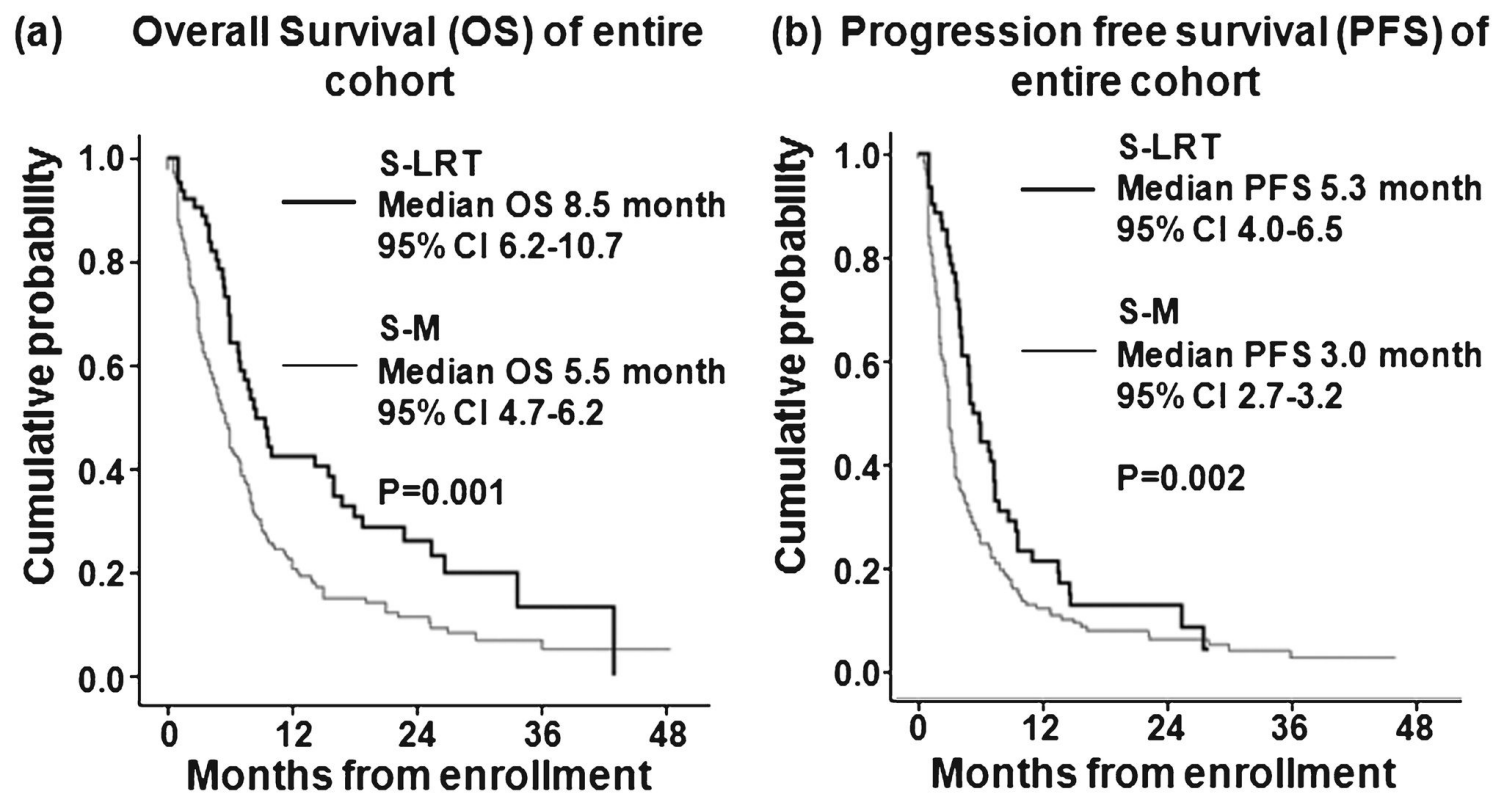
Kaplan-Meier analysis of overall survival (A) and progression-free survival (B) in patients Data were stratified by the treatment modalities treated with sorafenib monotherapy (S-M) and sorafenib-based loco-regional treatments (S-LRTs). The thick line indicates S-LRTs and the thin line S-M. Median OS and PFS are significantly longer in S-LRTs than S-M.

The median OS in the entire population was 6 months [95% confidence interval (CI) 5.2–6.7]. Subjects in the S-LRTs group had a significantly longer median OS than those in the S-M group [8.5 months (95% CI 6.2–10.7 months) vs. 5.5 months (95% CI 4.7–6.2 months); *P* = 0.001] (Figure 1). In addition, Child-Pugh class, tumor size, EHS and/or RNI, AFP level ≥400 ng/mL, PIVKA level ≥1,000 AU/L, and the cumulative dose of sorafenib (transformed by natural logarithm) significantly predicted OS in univariate analysis (all *P* < 0.05) (Table 2). Subsequent multivariate analysis revealed that combined LRTs modality with sorafenib remained as the independent predictor for the better OS [adjusted hazard ratio (HR) 0.5, 95% CI 0.3–0.8, *P* = 0.002], together with Child-Pugh class (adjusted HR 1.8, 95% CI 1.2–2.5, *P* < 0.001), tumor size (adjusted HR 1.5, 95% CI 1.1–2.3, *P* = 0.030), EHS and/or RNI (adjusted HR 1.7, 95% CI 1.2–2.4, *P* = 0.001), AFP level (adjusted HR 1.6, 95% CI 1.1–2.1, *P* = 0.002), and the cumulative dose of sorafenib (transformed by natural logarithm) (adjusted HR 0.5, 95% CI 0.4–0.6, *P* < 0.001 ) (Table 2).

**Table 2:** Univariate and multivariate analysis for variables affecting overall and progression-free survival.

**Variables**	**Overall survival**					**Progression-free survival**			
	**Univariate**		**Multivariate analysis**			**Univariate**		**Multivariate analysis**	
	***P***		**Adjusted HR (95% CI)**	***P***		***P***		**Adjusted HR (95% CI)**	***P***
S-LRTs (vs. S-M)	<0.001		0.5 (0.3–0.8)	0.002		0.002		0.6 (0.4–0.9)	0.025
Age ≥65 (vs. < 65 years)	0.819		-	-		0.116		-	-
Male (vs. female)	0.809		-	-		0.221		-	-
Viral etiology (vs. non-viral)	0.428		-	-		0.75		-	-
ECOG 0 (vs. 1/2)	0.944		-	-		0.297		-	-
Child-Pugh class B (vs. A)	<0.001		1.8 (1.2–2.5)	<0.001		0.003		1.4 (1.1–1.9)	0.029
Tumor size≥10 cm (vs. <10 cm)	<0.001		1.5 (1.1–2.3)	0.03		0.006		1.6 (1.1–2.4)	0.012
Presence of EHS and/or RNI (vs. no)	<0.001		1.7 (1.2–2.4)	0.001		<0.001		1.7 (1.2–2.4)	<0.001
Prior history of HCC treatment (vs. no)	0.949		-	-		0.992		-	-
AFP ≥400 ng/mL (vs. <400 ng/mL)	<0.001		1.6 (1.1–2.1)	0.002		<0.001		1.9 (1.4–2.5)	<0.001
PIVKA ≥1,000 AU/L (vs. <1,000 AU/L)	<0.001		1.2 (0.9–1.6)	0.161		<0.001		1.0 (0.7–1.3)	0.883
Ln total dosage (mg)	<0.001		0.5 (0.4–0.6)	<0.001		<0.001		0.6 (0.5–0.7)	<0.001

Abbreviations: HR, hazard ratio; CI, confidence interval; S-M, sorafenib monotherapy; S-LRTs, sorafenib combined with loco-regional treatments; ECOG, Eastern Cooperative Oncology Group; EHS, extrahepatic spread; RNI, regional nodal involvement; HCC, hepatocellular carcinoma; AFP, α-fetoprotein; PIVKA, protein induced by vitamin K absence; Ln, natural logarithm.

Because lung and/or bone metastasis significantly predicted OS in univariate analysis (*P* = 0.033), it was entered into multivariate analysis, adjusting other significant covariates such as S-LRTs, Child-Pugh class, tumor size, AFP level ≥400 ng/mL, PIVKA level ≥1,000 AU/L, and the cumulative dose of sorafenib. However, presence of EHS and/or RNI was not incorporated into this analysis since presence of EHS and/or RNI partly included lung and/or bone metastasis. Finally, lung and/or bone metastasis was also selected as one of the independent prognostic factor for OS (adjusted HR 1.2, 95% CI 1.1-1.8, *P* = 0.031) (Table S1).

### Treatment Outcomes and Variables Affecting PFS

The median PFS for the entire population was 3.4 months (95% CI 3.0–3.7). Subjects in the S-LRTs group had a significantly longer median PFS than those in the S-M group [5.3 months (95% CI 4.0–6.5 months) vs. 3.0 months (95% CI 2.7–3.2 months); *P* = 0.002; Figure 1]. In addition, Child-Pugh class, tumor size, EHS and/or RNI, AFP level ≥400 ng/mL, PIVKA level ≥1,000 AU/L, and the cumulative dose of sorafenib (transformed by natural logarithm) significantly predicted PFS in univariate analysis (all *P* < 0.05; Table 2). Subsequent multivariate analysis revealed that combined LRTs modality with sorafenib remained as the independent predictor for the better PFS (adjusted HR 0.6, 95% CI 0.4–0.9, *P* = 0.025), together with Child-Pugh class (adjusted HR 1.4, 95% CI 1.1–1.9, *P* = 0.029), tumor size (adjusted HR 1.6, 95% CI 1.1–2.4, *P* = 0.012), EHS and/or RNI (adjusted HR 1.7, 95% CI 1.2–2.4, *P* < 0.001 ), AFP level (adjusted HR 1.9, 95% CI 1.4–2.5, *P* < 0.001), and the cumulative dose of sorafenib (transformed by natural logarithm) (adjusted HR 0.6, 95% CI 0.5–0.7, *P* < 0.001) (Table 2).

Because lung and/or bone metastasis significantly predicted PFS in univariate analysis (*P* = 0.001), it was entered into multivariate analysis, adjusting other significant covariates such as S-LRTs, Child-Pugh class, tumor size, AFP level ≥400 ng/mL, PIVKA level ≥1,000 AU/L, and the cumulative dose of sorafenib. However, presence of EHS and/or RNI was not incorporated into this analysis since presence of EHS and/or RNI partly included lung and/or bone metastasis. Finally, lung and/or bone metastasis was also selected as one of the independent prognostic factor for PFS (adjusted HR 1.5, 95% CI 1.1-2.1, *P* = 0.005) (Table S1).

### Subgroup Analysis According to EHS or Regional Node Metastasis and AFP

The OS and PFS of subgroups according to the presence of EHS and/or RNI were compared (Table 3). In each subgroup, patients treated with S-LRTs had longer OS compared to those treated with S-M (18.0 months vs. 7.8 months in a subgroup with neither EHS nor RNI and 8.3 months vs. 4.8 months in a subgroup with EHS and/or RNI; all *P* < 0.05). In addition, patients treated with S-LRTs had longer PFS compared to those treated with S-LRTs in a subgroup with neither EHS nor RNI (9.6 months vs. 3.2 months, *P* = 0.027). However, the therapeutic benefit of LRTs was only marginal in a subgroup with EHS and/or RNI (4.9 months vs. 2.9 months, *P* = 0.069).

**Table 3:** Overall and progression-free survival according to tumor factors in subgroups.

**Population**		**Median OS (95% CI) (months)**		**Hazard Ratio (95% CI) for S-LRTs (vs. S-M)**	***P***
		**S-M**	**S-LRTs**		
*Entire cohort*		5.5 (4.7–6.2)	8.5 (6.2–10.7)	0.5 (0.4–0.7)	0.001
	Subgroup with neither EHS nor RNI	7.8 (6.2–9.3)	18.0 (3.7–33.2)	0.4 (0.2–0.9)	0.019
	Subgroup with EHS and/or RNI	4.8 (3.7–5.8)	8.3 (5.0–11.5)	0.6 (0.4–0.9)	0.028
	Subgroup with AFP <400 ng/mL	7.8 (6.6–8.9)	8.3 (5.2–11.3)	0.8 (0.5–1.3)	0.449
	Subgroup with AFP ≥400 ng/mL	3.4 (2.5–4.2)	8.0 (0.0–19.0)	0.3 (0.1–0.6)	0.001
**Population**		**Median PFS (95% CI) (months)**		**Hazard Ratio (95% CI) for S-LRTs (vs. S-M)**	***P***
		**S-M**	**S-LRTs**		
*Entire cohort*		3.0 (2.7–3.2)	5.3 (4.0–6.5)	0.6 (0.4–0.8)	0.002
	Subgroup with neither EHS nor RNI	3.2 (1.8–4.5)	9.6 (3.1–16.0)	0.5 (0.2–0.9)	0.027
	Subgroup with EHS and/or RNI	2.9 (2.5–3.2)	4.9 (4.0–5.7)	0.7 (0.4–1.0)	0.069
	Subgroup with AFP <400 ng/mL	4.0 (2.6–5.3)	6.8 (4.1–9.4)	0.7 (0.4–1.1)	0.225
	Subgroup with AFP ≥400 ng/mL	2.0 (1.6–2.3)	4.2 (2.6–5.7)	0.5 (0.3–0.8)	0.012

Abbreviations: OS, overall survival; PFS, progression-free survival; CI, confidence interval; S-M, sorafenib monotherapy; S-LRTs, sorafenib combined with loco-regional treatments; EHS, extrahepatic spread; RNI, regional nodal involvement.

 The OS and PFS of subgroups were also affected by AFP level (<400 ng/mL vs. ≥400 ng/mL). In the subgroup with low AFP level (<400 ng/mL), the therapeutic benefit of LRTs to prolong OS did not reach the statistical significance (*P* = 0.449). However, in the subgroup with high AFP level (≥400 ng/mL), the LRTs was effective to improve the OS (3.4 months vs. 8.0 months, *P* = 0.001). Regarding PFS, similar results were obtained. Although the S-LRTs could not increase PFS in the subgroup of low AFP level (<400 ng/mL) (*P* = 0.225), it extended PFS significantly in the subgroup of high AFP level (≥400 ng/mL) (2.0 months vs. 4.2 months, *P* = 0.012).

### Treatment-Related Toxicity

The treatment-related adverse effects of grade 2 or more are described in Table 4. The most common toxicity related to sorafenib was hand foot-skin reaction (23.0% in S-M group vs. 23.4% in S-LRTs group), followed by diarrhea (16.4% in S-M group vs. 15.6% in S-LRTs group). Grade 3 or 4 toxicities were relatively uncommon and hand foot-skin reaction of grade 3 or more was found in 8 (4.8%) patients in the S-M group and 2 (3.7%) patients in the S-LRTs group. Except anorexia, the addition of LRTs to sorafenib did not increase the occurrence of significant toxicities (all *P* > 0.05). All adverse events were manageable and there was no significance difference of the sorafenib discontinuation due to adverse effects between two groups. 

**Table 4:** Treatment-related adverse effects.

		**S-M**	**S-LRTs**	***P***
Major adverse effects of grade 2 or more				
	HFSR	52 (23.0)	15 (23.4)	NS
	Diarrhea	37 (16.4)	10 (15.6)	NS
	Skin eruption	17 (7.5)	7 (10.9)	NS
	Anorexia	10 (4.4)	8 (12.5)	0.034
	Abdominal pain	19 (8.4)	8 (12.5)	NS
	Sorafenib discontinuation due to adverse effects	64 (28.3)	19 (29.7)	NS

Abbreviations: S-M, sorafenib monotherapy; S-LRTs, sorafenib combined with loco-regional treatments; HFSR, hand foot-skin reaction; NS, not significant.

## Discussion

For advanced-stage disease, the multikinase inhibitor sorafenib is the new standard treatment with a proven survival benefit [5,6]. However, since sorafenib predominantly results in delayed tumor progression by inhibiting tumor cell proliferation rather than shrinking tumors, most patients treated with sorafenib alone achieve only stable disease as the best radiologic response, with a median time to progression of from 2.2 to at most 5.5 months. Thus, new methods beyond sorafenib treatment are urgently required for HCC. In contrast, active LRTs including intra-arterial chemotherapy (TACE or HAIC) and/or external beam radiotherapy induce complete or partial radiologic response of approximately 30%, even in advanced HCC [22,24,26,27]. Considering that more than two-thirds of patients with advanced HCC die of liver failure due to intrahepatic tumor progression rather than from progression of extrahepatic metastatic disease, addition of such radical LRTs targeting the primary tumor might be a reasonable and effective approach for treating advanced-stage HCC, even in the presence of MVI, regional lymph node metastasis or EHS. To obtain pilot data, we investigated the benefits of providing active LRTs with sorafenib to patients with advanced-stage HCC.

 The OS of the S-M group in our study was relatively shorter than that of Asia-Pacific trial (5.5 months vs. 6.5 months) [6]. This can be explained in part by the higher proportion of patients with older age (median 56 years vs. 51 years), poorer liver function (Child-Pugh class B, 28.3% vs. 2.7%), and more advanced stage of HCC (BCLC C, 100% vs. 95.3%) in our study population. Nevertheless, in our study S-LRTs prolonged the median OS by up to 3 months (8.5 months, *P* = 0.001), compared to that of the S-M group (5.5 months). This benefit of combined treatment was also confirmed through multivariate analysis after adjusting for other predictors such as Child-Pugh class, tumor size, EHS and/or RNI, alpha-fetoprotein level, and cumulative dosage of sorafenib. The beneficial effects of S-LRTs on OS were similarly observed in subgroups of patients with neither EHS nor RNI and those with EHS and/or RNI. Likewise, regarding PFS, S-LRTs prolonged the median PFS by up to 2.3 months (*P* = 0.002). Furthermore, when lung and/or bone metastasis were incorporated into multivariate analysis instead of EHS and/or RNI, the independent prognostic values of lung and/or bone metastasis and S-LRT were also similarly maintained for both OS and PFS.

Interestingly, when tumor burden was high, reflected by high AFP level (≥400 ng/mL), the effectiveness of S-LRTs became more prominent in improving OS and PFS (Table 3). These results support again the rationale that LRTs should be considered for advanced HCC with high tumor burden. Taken together, S-LRTs might delay intra-hepatic tumor progression and by extension, result in preserving the remnant liver function and ultimately prolonging the OS. The mechanism of action remains to be further investigated. 

 Although the promising results of active LRTs warrant further validation in larger prospective trials, our study had several strengths. First, the sample size in this study was larger and the follow-up period was longer than any previous studies [18-20]. Second, we have suggested how to improve the treatment responses of sorafenib treatment on the assumption that active LRTs might delay the hepatic failure due to the intrahepatic tumor progression. Thus, this study might be expected to provide a basic reference for further research on the addition of LRTs with sorafenib administration. Third, we performed subgroup analysis according to tumor status in order to identify who are more likely to benefit from additional LRTs. The therapeutic benefit for both OS and PFS was greater in the subgroup with neither EHS nor RNI than in the subgroup with EHS and/or RNI. However, since additional LRTs are aimed primarily at controlling the disease progression in the liver, the therapeutic effect for prolonging PFS is only marginal in a certain subgroup with EHS and/or RNI where the progression of EHS or RNI is beyond the effect of additional LRTs. Strikingly, even in such a subgroup, additional LRTs were helpful in reducing the events of hepatic failure due to intrahepatic tumor progression, ultimately leading to significantly prolonged OS. Therefore, the concurrent use of active LRTs might be a reasonable approach for treating advanced HCC. 

 Notably, among other predictors, the cumulative dosage of sorafenib, which depends on the daily administration dosage and duration of treatment, proved to be an independent prognostic predictor for both OS and PFS. This observation indicates that sorafenib should be administered as long as patients tolerate the treatment and further confirms the importance of sorafenib at the core of HCC therapy. In the current study the incidence of adverse effects due to sorafenib treatment were similar to those reported in other investigations [28-30]. More importantly, the addition of active LRTs did not increase the overall incidence of adverse events owing to sorafenib administration. However, physicians should always exercise caution when combining modalities.

 There are several possible explanations for the beneficial effects of combining sorafenib with other LRTs. Although TACE induces tumor hypoxia, angiogenic factors such as VEGF temporarily increase after TACE. Therefore, the enhanced expression of circulating or tissue VEGF after TACE treatment could adversely affect the outcome of HCC patients, through revascularization, tumor progression, and distant metastasis [31]. From this view point, combining an anti-angiogenic agent with TACE may provide complementary inhibition of neovascularization and tumor growth [32,33]. Similarly, radiation exposure may act as a stressing event, inducing the compensatory activation of multiple intracellular signaling pathway mediators, such as phosphoinositide 3-kinase, mitogen-activated protein kinase (MAPK), c-Jun N-terminal kinase, and nuclear factor-kappa B [34,35]. In particular, VEGF levels increased in a time- and dose-dependent manner after sublethal irradiation of HCC cells, which translated to enhanced intratumor angiogenesis in vivo [36]. Therefore, sorafenib-mediated blockage of the Raf/MAPK and vascular endothelial growth factor receptor pathways might enhance the efficacy of radiation [37,38].

 This study has a few limitations. The first drawback is the retrospective nature of the work, which could lead to selection bias in determining the treatment modalities. However, we consecutively enrolled subjects during the study period and the baseline characteristics between the two groups were very similar, differing only in the proportion of HCCs with viral etiologies and previous history of treatment for HCC. As a matter of fact, the etiologies for HCC and prior treatment history did not affect the clinical outcomes. Second, active LRTs used in this study include heterogeneous modalities such as TACE, HAIC, external-beam radiotherapy, and their combinations. Although such LRTs demonstrate comparable outcomes [15,22,34,39], prospective trials are required to solve this issue. In addition, treatment regimen decisions were based not only on medical issues, but also non-medical and/or economic considerations. 

 In conclusion, the addition of active LRTs to sorafenib treatment achieved promising results in unresectable HCC without significantly increasing treatment-related toxicities. Further, the therapeutic benefits for OS were maintained regardless of the presence of EHS. These results now require validation in another population through prospective trials.

## Supporting Information

Table S1 **Univariate and multivariate analysis for variables including lung and/or bone metastasis to affect overall and progression-free survival.**
(DOC)
