# Supplementary material for: Efficacy of Sorafenib Monotherapy versus Sorafenib-Based Loco-Regional Treatments in Advanced Hepatocellular Carcinoma
Source: PLoS One. 2013 Oct 14;8(10):e77240. doi: 10.1371/journal.pone.0077240 (PMC3796498; doi:10.1371/journal.pone.0077240)
Supplement: Table S1 — Univariate and multivariate analysis for variables including lung and/or bone metastasis to affect overall and progression-free survival. (DOC) [file pone.0077240.s001.doc]

| **Variables** | **Overall survival** | | | |  | **Progression-free survival** | | | |
| --- | --- | --- | --- | --- | --- | --- | --- | --- | --- |
|  | **Univariate** |  | **Multivariate analysis** | |  | **Univariate** |  | **Multivariate analysis** | |
|  | ***P*** |  | **Adjusted HR (95% CI)** | ***P*** |  | ***P*** |  | **Adjusted HR (95% CI)** | ***P*** |
| S-LRTs (vs. S-M) | <0.001 |  | 0.5 (0.3–0.7) | 0.002 |  | 0.002 |  | 0.6 (0.4–0.9) | 0.024 |
| Child-Pugh class B (vs. A) | <0.001 |  | 1.8 (1.3–2.5) | <0.001 |  | 0.003 |  | 1.4 (1.1–1.9) | 0.024 |
| Tumor size≥10 cm (vs. <10 cm) | <0.001 |  | 1.5 (1.1–2.4) | 0.028 |  | 0.006 |  | 1.6 (1.1–2.3) | 0.014 |
| Lung and/or bone metastasis (vs. no) | 0.033 |  | 1.2 (1.1-1.8) | 0.031 |  | 0.001 |  | 1.5 (1.1-2.1) | 0.005 |
| AFP ≥400 ng/mL (vs. <400 ng/mL) | <0.001 |  | 1.6 (1.1–2.1) | 0.003 |  | <0.001 |  | 1.9 (1.4–2.6) | <0.001 |
| PIVKA ≥1,000 AU/L (vs. <1,000 AU/L) | <0.001 |  | 1.2 (0.9–1.6) | 0.161 |  | <0.001 |  | 1.0 (0.7–1.3) | 0.883 |
| Ln total dosage (mg) | <0.001 |  | 0.5 (0.4–0.6) | <0.001 |  | <0.001 |  | 0.6 (0.5–0.7) | <0.001 |

**Table S1.** Univariate and multivariate analysis for variables including lung and/or bone metastasis to affect overall and progression-free survival.

Abbreviations: HR, hazard ratio; CI, confidence interval; S-M, sorafenib monotherapy; S-LRTs, sorafenib combined with loco-regional treatments; ECOG, Eastern Cooperative Oncology Group; HCC, hepatocellular carcinoma; AFP, α-fetoprotein; PIVKA, protein induced by vitamin K absence; Ln, natural logarithm.
